# Predicting the Diagnostic Information of Tandem Mass Spectra of Environmentally Relevant Compounds Using Machine Learning

**DOI:** 10.1021/acs.analchem.3c03470

**Published:** 2023-10-09

**Authors:** S. Codrean, B. Kruit, N. Meekel, D. Vughs, F. Béen

**Affiliations:** † Faculty of Science, Artificial Intelligence, Vrije Universiteit Amsterdam, De Boelelaan 1085, 1081 HV Amsterdam, The Netherlands; ‡ KWR Water Research Institute, Groningenhaven 7, P.O. Box 1072, 3430 BB Nieuwegein, The Netherlands; § Chemistry for Environment and Health, Amsterdam Institute for Life and Environment (A-LIFE), Vrije Universiteit De Boelelaan 1085, 1081 HV Amsterdam, The Netherlands

## Abstract

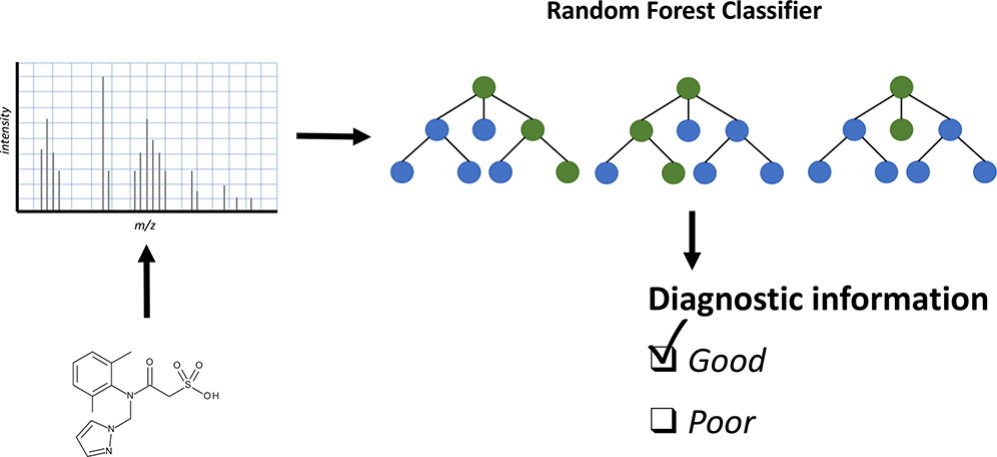

Acquisition and processing of informative tandem mass spectra (MS2) is crucial for numerous applications, including library-based (tentative) identification, feature prioritization, and prediction of chemical and toxicological characteristics. However, for environmentally relevant compounds, approaches to automatically assess the quality of the MS2 spectra are missing. This work focused on developing a machine learning-based approach to automatically evaluate the diagnostic information of MS2 spectra (e.g., number, distribution, and intensity of diagnostic fragments) of environmentally relevant compounds analyzed with electrospray ionization. For this, approximately 1400 MS2 spectra of 204 environmental contaminants, acquired with different collision energies using liquid chromatography coupled to high-resolution mass spectrometry, were used to train a random forest classifier to distinguish between spectra providing *good* or *poor* diagnostic information. Prior to training, validation, and testing, spectra were manually labeled based on criteria such as number, intensity, range of fragments present, molecular ion intensity, and noise levels. Subsequently, feature engineering and selection were applied to retrieve relevant variables from raw MS2 spectra as inputs for the classifier. The optimal set of features based on model performances was selected and used to train a final model, which showed an accuracy of 84%, a precision of 88%, and a recall of 75%. Results show that the combination of selected features and the machine learning model used here can effectively distinguish between MS2 spectra providing *good* or *poor* diagnostic information according to the defined criteria. The developed model has the potential to improve a broad range of applications that rely on MS2 data.

## 1 Introduction

High-resolution mass spectrometry (HRMS) coupled with either liquid (LC) or gas chromatography (GC) has become an essential tool to monitor emerging contaminants in the environment.^1^ In particular, the acquisition of tandem mass spectrometry (MS2) spectra combined with the ever growing quality and comprehensiveness of spectral libraries (e.g., MassBankEU,^2^ MoNA^3^) have greatly expanded the possibilities offered by suspect and nontarget screening analyses.^4,5^ Despite continuous improvements, large discrepancies still exist between the number of potentially relevant contaminants present in environmental samples and those for which spectral information is available in libraries.^6^ Moreover, despite the development of workflows to automatically improve the quality of records added to these libraries,^7^ and the acquisition of multiple spectra per compound to account for specific fragmentation curves, issues regarding quality assurance and control (QA/QC) of the information contained in these databases still exist, including insufficiently curated tandem mass spectra.^6,8^ In the field of proteomics, where database searches and *de novo* sequencing approaches are used to identify peptides from complex mixtures of proteins,^9−11^ the quality of tandem mass spectra, and how to assess it, have been the subject of various studies. In this context, spectral quality should be understood as the amount of diagnostic information about the structure of the parent ion provided by the MS2 spectra. Specifically, these should have sufficient diagnostic fragments spread across the whole mass range (relative to the mass of the parent ion) and with sufficient intensity as well as little to no noise. In fact, for library-based peptide identifications, poor MS2 data quality is considered to play a major role in the occurrence of false negatives.^9^ For this purpose, already in the early 2000s, algorithms have been devised to try to automatically assess the quality of MS2 spectra acquired in proteomics experiments.^12^ Recently, more advanced machine and even deep learning algorithms have been developed to automatically assess the quality of acquired MS2 signals, reduce the occurrence of false negatives, and decrease overall processing time of large data sets.^9,13,14^ The proposed classifiers showed very promising results. For instance, the approach developed by Bern et al.^12^ was able to eliminate over 75% of spectra considered as being of bad quality and, at the same time, would only lose 10% of spectra deemed as being of good quality. Using support vector machine (SVM) and k-means, Zou et al.^14^ and Ding et al.^13^ were able to develop binary classifiers having true positive rates (TPR) of 92% and 90% while keeping the true negative rate (TNR) at 90% and 92%, respectively. These methods often rely on a range of “features” (to be understood here as descriptors, or independent variables, rather than HRMS-based features) derived from peptide fragmentation patterns, such as b- and y-ion peaks^15^ or amino acid sequence tags.^11^ Only more recently, a deep learning method was developed which takes the entire MS2 spectrum (after preprocessing and normalization) to assess spectral quality.^9^ The fact that most models developed so far used features derived from specific peptide fragmentation patterns, combined with the difficulty to objectively establish criteria to define an MS2 spectrum providing diagnostic information, might explain why these approaches have not yet been implemented in other fields. In fact, in the specific case of (small) environmentally relevant molecules, the issue of MS2 diagnostic information has not been addressed thoroughly, besides in the general context of curating spectral libraries and the development of search and matching algorithms.^6^ Yet, obtaining MS2 spectra providing diagnostic information could improve both feature annotation and reduce overall (post)processing time in environmental analyses. However, the importance of obtaining high-quality MS2 spectra is not limited to annotations or library searches. In fact, in recent years, an increasing number of computational tools have been reported that make use of MS2 data to improve postprocessing and prioritization (e.g., molecular networking strategies^16,17^), predict molecular structures,^18^ or even *in vivo* toxicity end points of unknowns.^19,20^ Given that these methods rely on MS2 spectra, their performances would most likely benefit from having input data of high(er) quality. Furthermore, algorithms used to determine the quality of MS2 spectra could in the future be integrated into data-dependent acquisition (DDA) methods and used to determine if acquired spectra provide sufficient diagnostic information or if additional ones (e.g., different collision energy (CE)) should be recorded. While for data-independent acquisition (DIA), such information could be useful during postprocessing to prioritize MS2 spectra rich in diagnostic information.

The goal of this work was hence to develop a machine learning pipeline to automatically assess the diagnostic information of electrospray ionization (ESI) MS2 spectra of environmentally relevant compounds. For this purpose, a data set of 204 reference standards of environmental contaminants acquired with different CEs, corresponding to almost 1400 MS2 spectra, was used. Initially, the focus was set on finding relevant features (i.e., descriptors used for modeling purposes) that could be used for machine learning purposes and that provided a sufficiently accurate representation of the raw input data. Specifically, three different feature sets were computed, and their performances were evaluated using a random forest (RF) classifier with cross-validation. Computed descriptors were then further filtered to select those that explained most of the available data. Finally, the optimized feature sets were evaluated against the test set and the model’s classification threshold was set to favor precision and reduce false positives.

## 2 Experimental Section

### 2.1 Data Set

The data set used in this work consisted of fragmentation mass spectra (MS2) of 204 reference standards of known environmental contaminants (see the Supporting Information for a complete list) which were analyzed by liquid chromatography (LC) coupled to an Orbitrap Fusion Tribrid high-resolution mass spectrometry instrument (HRMS, Thermo Fisher Scientific) equipped with a heated electrospray ionization source. Separation was achieved using a generic chromatographic method using an XBridge BEH C18 (2.5 μm, 2.1 × 100 mm Column XP, Waters) column as described in Been et al.^21^ Acquisition was performed in data-dependent acquisition (DDA) mode with high collision dissociation (HCD) and graded collision energy (CE) of 10, 20, 35, 50, 65, 80 and 100%. MS2 spectra obtained were then searched using the retention time of each reference standard and by retrieving the scan corresponding to each of the CEs used. Spectra were acquired in profile mode but were then converted to centroids to facilitate comparison with existing spectra libraries. The final data set consisted of 1399 MS2 spectra.

### 2.2 Initial Labeling of MS2 Spectra

Initially, labeling of acquired MS2 spectra was carried out automatically. More specifically, matching spectra were searched in MassBankEU^22^ using the *SpectrumSimilarity* function from the OrgMassSpecR package developed by Dodder and Mullen.^23^ This was done after preliminary filtering of spectra based on precursor masses. Spectra eliciting a high score (≥0.75) were initially labeled as being *good* while spectra with lower scores were labeled as *poor*. However, due to the differences in both fragmentation approaches and collision energies (CEs) used, inconsistencies were observed in the labeling. In particular, spectra were incorrectly labeled. Because of the difficulty of defining quantitative criteria which could be used to automatically label MS2 spectra, it was decided to rely on expert judgment and to manually label all spectra. To mitigate the subjectivity of the labeling step, a set of qualitative criteria that spectra had to satisfy to be labeled as *good* was defined:

(i) Number of diagnostic fragments: at least 2 diagnostic fragments are present in the spectrum. Diagnostic fragments should provide relevant structural information, for instance, losses of specific structures/functionalities (e.g., carboxylic acid or ester [M+H^+^-44 or aromatic groups [M+H^+^-77]) in contrast to nonspecific ones (e.g., loss of water [M+H^+^-18] or a methyl group [M+H^+^-15]).
(ii) Intensity of diagnostic fragments: diagnostic fragments should be present at an intensity >5% of the base peak in the MS2 spectrum.
(iii) Fragment distribution: diagnostic fragments should be spread over the whole *m*/*z* range (i.e., from the lower limit up to the *m*/*z* of the precursor ion/adduct).
(iv) Precursor intensity: the precursor intensity should not exceed the 25th percentile of the intensity distribution of diagnostic fragments.
(v) Noise level: diagnostic fragments should be clearly distinguishable from the noise.

Following the labeling process, a subset of spectra was randomly selected to verify the correctness of the labeling procedure. The final data set consisted of 1399 MS2 spectra, of which 615 (44%) and 784 (56%) were labeled as containing *good* and *poor* diagnostic information, respectively.

### 2.3 Preprocessing

**Figure 1:**
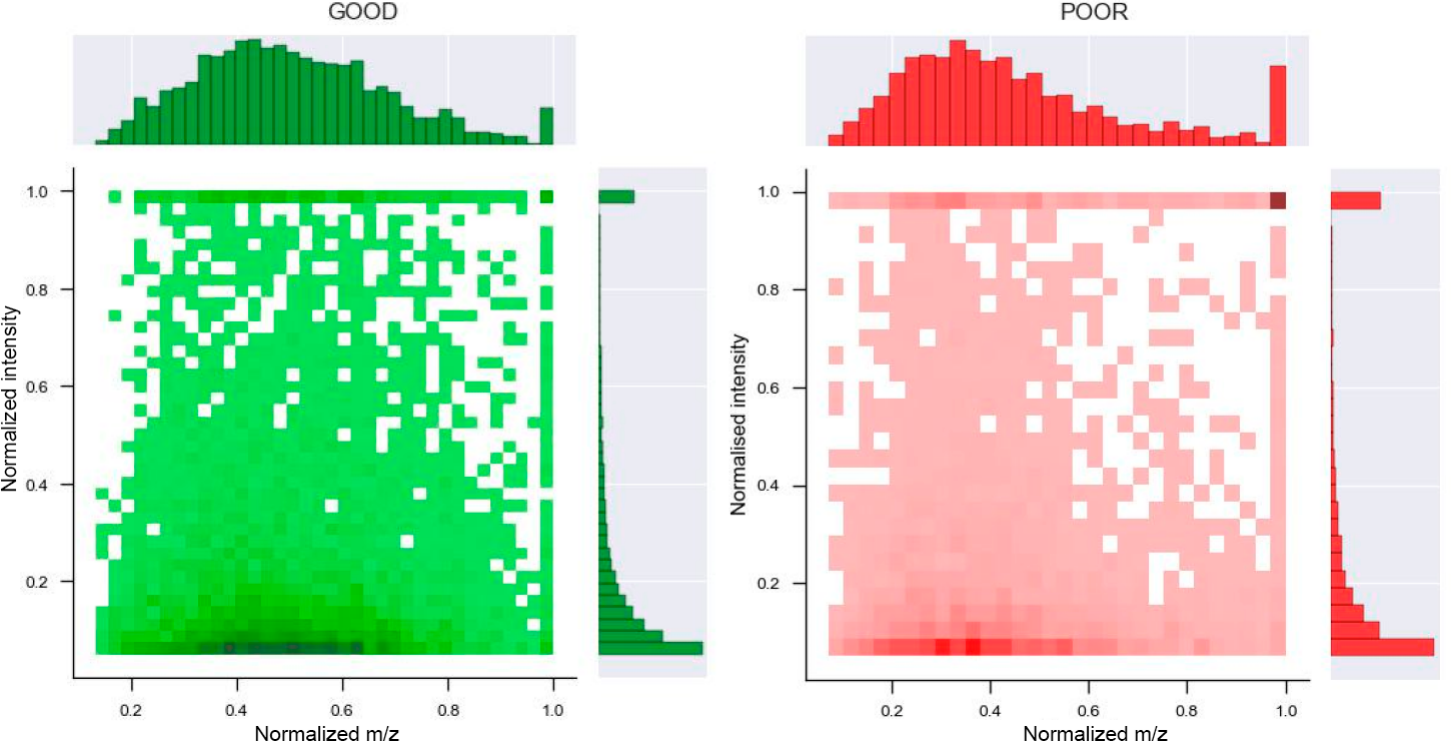
Distribution of normalized *m*/*z* and intensities in MS2 spectra labeled as good (left) and poor (right). *m*/*z* values were normalized according to the *m*/*z* of the precursor. Intensities were normalized through the most intense/base peak in the MS2 spectrum.

Prior to calculating features (i.e., descriptors), MS2 spectra were scaled with respect to both their intensity and the *m*/*z* range. Relative intensities (i.e., range [0, 1]) were computed by dividing individual intensities by the intensity of the base peak. Similarly, the *m*/*z* range of each spectrum was normalized by dividing individual *m*/*z* values by the *m*/*z* value of the precursor. Finally, noise was removed by filtering all *m*/*z* whose intensity was ≤5% of the base peak. An overview of the distribution of the preprocessed MS2 spectra is shown in Figure 1.

### 2.4 Feature Transformation

#### 2.4.1 Distance Features

The first set of features which were computed consisted of statistics derived from the calculation of Euclidean distance between the centroid of each spectrum and the remaining *m*/*z* after preprocessing. The centroid *c* was defined as follows:
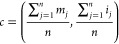
1where *m*_*j*_ are the *m*/*z* values in the spectrum, *i*_*j*_ are the corresponding intensities, and *n* is the number of *m*/*z* values in the spectrum. For every *m*/*z* value (*p*) in the spectrum, the Euclidean distance *d* to the centroid *c* is calculated by the formula
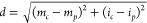
2where *m*_*p*_ and *i*_*p*_ are the *m*/*z* and corresponding intensity of the *p*th *m*/*z* value in the spectrum, while *m*_*c*_ and *i*_*c*_ are the *m*/*z* and intensity of the centroid. Using the distance vector, the count; mean; standard deviation; minimum; maximum; and first, second, and third quartiles were calculated and used as *distance features* for modeling purposes (see Table S1).

#### 2.4.2 Handcrafted Features

The second set of features computed from MS2 spectra consists of a collection of common features found in the literature, together with some empirically selected features. Specifically, the number of *m*/*z* values in the spectrum,^11,12,14,24^ the average,^11^ sum^24^ and standard deviation of intensities in each spectrum were computed. Additionally, the dot product between *m*/*z* and intensity values was also used as a feature. The number of peaks with relative intensity greater than 0.1^14^ and 0.2 were also considered. The standard deviation of the difference in *m*/*z* between consecutive fragments and the average number of fragments in a 2 Dalton (Da) interval^11^ were also used. The intensity balance, calculated by dividing the *m*/*z* axis into a number of bins of equal width and subtracting the total intensity of the first bin from the sum of the intensities of the remaining bins,^12^ the entropies for the *m*/*z* and intensity vectors were also used (see Table S1).

#### 2.4.3 Grid Features

Inspired by previous work from Logan et al.,^25^ the last feature set consisted of dividing the spectra into 1- or 2-dimensional (1D or 2D) grids and counting the number of points (i.e., *m/z*) in each grid cell. In 1D-grids, between 1 and 20 bins were unevenly distributed along the intensity (*y*) axis to have more granularity (i.e., more frequent bins) at lower intensities compared to higher ones. In the case of 2D-grids, between 1 and 20 bins were considered both for the *m*/*z* (*x*) and the intensity (*y*) axis (i.e., yielding *N* × *N* matrices; see Table S1).

### 2.5 Statistical Modeling

In the context of this work, a random forest (RF) algorithm^26^ was used given its widespread use in the context of binary classification,^27,28^ and the fact that it is often considered the method of choice with expected highly nonlinear relationships. The RF was trained using the *RandomForestClassifier* function from the *sklearn.ensemble* module in Python. The training involved bootstrapping, “balanced” *class_weights*, a “fixed” *random_state* and *max_features = “sqrt”*, while the function *GridSearchCV* was used to tune *n_estimators: [50, 100, 200]*, *max_depth: [None, 10, 20, 30]*, *min_samples_split: [2, 5, 10]*, and *min_sample_leaf: [1, 2, 4]*. The CE and precursor *m*/*z* were added to each of the above-mentioned feature sets. Prior to training, validation, and testing, features were centered and scaled. Features’ importance was retrieved for each set using the *feature_importances_* (i.e., impurity-based) property of the *RandomForestClassifier* function. The raw MS2 data used and Python code used to compute the feature sets and RF models can be found at https://github.com/svetlanacodrean/HRMS-Quality-assessment. Readers interested in additional information about the developed algorithms should contact the authors directly.

### 2.6 Validation and Testing

The initial data set of 1399 MS2 spectra was divided into two parts, namely, 949 (67.8%) observations for training and 450 (32.2%) observations were kept for final testing. Both sets had 44% instances labeled as good and 56% labeled *poor*. Feature groups were evaluated individually, and then results were compared. Feature groups were evaluated by applying a stratified 10-Fold Cross-Validation^29^ over the 949 instances provided for training, resulting in 10 training iterations with 854 instances for fitting and 95 samples for prediction. Metrics used to evaluate model performances were the iteration accuracy, average precision, Area Under the Receiver Operating Characteristic Curve (ROC AUC) score, and log loss of the model. In the context of this work, particular attention was given to precision given that it was considered that the impact of misclassifying a poor spectrum as *good* would be greater than vice versa. In fact, such a misclassification could lead to unfruitful and time-consuming (tentative) identification of features (referred to here as a “chemical feature” resulting from HRMS analysis) having poor MS2 data or performing any other type of statistical analysis based on an MS2 spectrum of insufficient quality. Similarly, should this kind of classification algorithm be implemented during acquisition (i.e., determining whether an additional MS2 spectrum needs to be acquired in a DDA experiment), a conservative strategy would entail recording an additional MS2 spectrum, even if the existing one already contained valuable diagnostic information, rather than relying on a spectrum of lower quality. For *Grid* features, feature selection focused on finding the optimal number of 1D and 2D bins. *Handcrafted* and *Distance* feature sets were evaluated individually and combined. A Spearman rank correlation test was applied to the combined set, and features having a dissimilarity ≥0.3 were marked as *uncorrelated* and evaluated once more separately. *Grid* features were not included in the correlation testing because their structure is inherently different, while both *Distance* and *Handcrafted* features are based on heuristics and are likely going to contain similar information because criteria for computing them were partially similar. *Handcrafted* and *Distance* features (and the combination thereof) were also evaluated using cross-validated recursive feature elimination (RFECV).^30^ Finally, during the testing phase, a simple baseline model (i.e., a random forest classifier trained with only the number of fragments present in the MS2 spectrum) was used to evaluate the described feature extraction methods and get an idea of the expected model performance.

## 3 Results and Discussion

### 3.1 Grid Feature Selection

**Figure 2:**
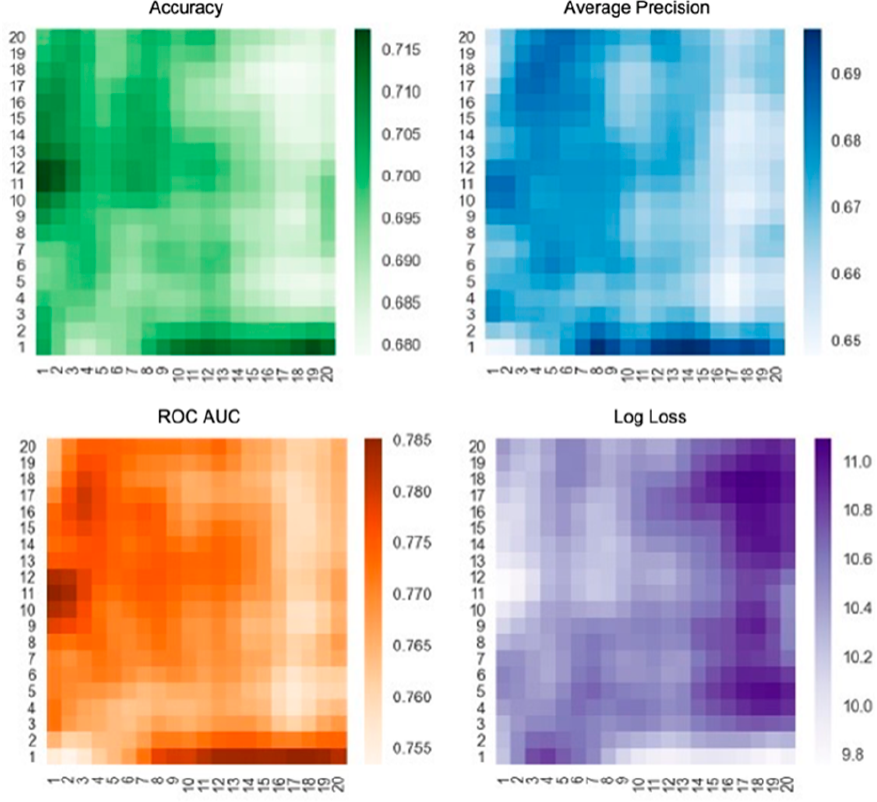
Metrics for each combination of numbers of *m*/*z* (*x*-axis) and intensity (*y*-axis) bins in 2D. Each value represents the average metric score (together with the standard deviation) obtained from a stratified 10-fold cross-validation for a given (#m/z, #intensity) combination. A Gaussian blur filter (σ = 1) was applied to the heatmap to facilitate the visualization of the results.

Prior to evaluating model performances on the holdout (test) set, the optimal number of bins in both the 1D (unevenly distributed) and the 2D grids were evaluated. First, the optimal grid specification was searched, namely, the number of bins per axis (*m*/*z* and intensity) from which the 2D distribution of the *m*/*z*-intensity pairs is obtained. From this distribution, specified by the number of bins on each axis, *N* × *N* features were derived as described previously. Combinations of *m*/*z* and intensity bins from 1 to 20 were evaluated. It is worth mentioning that pair (1, 1) means that there is only one bin for the *m*/*z* values and one bin for the intensity values and hence corresponds to the number of fragments in a spectrum. The heatmaps in Figure 2 show the results for all metrics. From a first observation, it appears that the use of a highly granular grid does not provide particularly good results, as lower performances are obtained when a large number of bins are used to divide the *y*- and *x*-axes (Figure 2). A closer look reveals an almost identical pattern in all four metrics. Areas with highest scores (i.e., darkest shades) are in two locations in the 2D space. In the case of log loss, it is the opposite, as one seeks to obtain the smallest metric. Visual inspection indicates that the best-performing pairs are (*m*/*z*, 1), ∀× ∈ {8, 9,···, 20}, but also the pairs (*m*/*z*, *intensity*), *m*/*z* ∈ {1,2}, *intensity* ∈ {11,12}. These results suggest that the use of 1D histograms is preferable to that of 2D histograms. One possible explanation could be that the more granular the space becomes, the sparser the grid cells (i.e., most values are equal to zero). The four best bin combinations for the 2D grid, namely, (19,1), (10,1), (12,1), and (2,11), were selected for further comparisons (see Table S2 for all details). Results obtained using the 1D unevenly distributed grid are shown in Figure S1. In this specific case, no difference was observed as the number of bins was increased up to 20, suggesting that the granularity of the lowest layers does not play an important role, likely because noise (i.e., *m*/*z* values having an intensity <5% of the maximum) was removed during preprocessing. Nevertheless, the best-performing bin dimension was 14 (i.e., accuracy of 71%, average precision of 68%, ROC AUC 77% and log loss of 9.93).

### 3.2 Feature Selection and Validation

**Figure 3:**
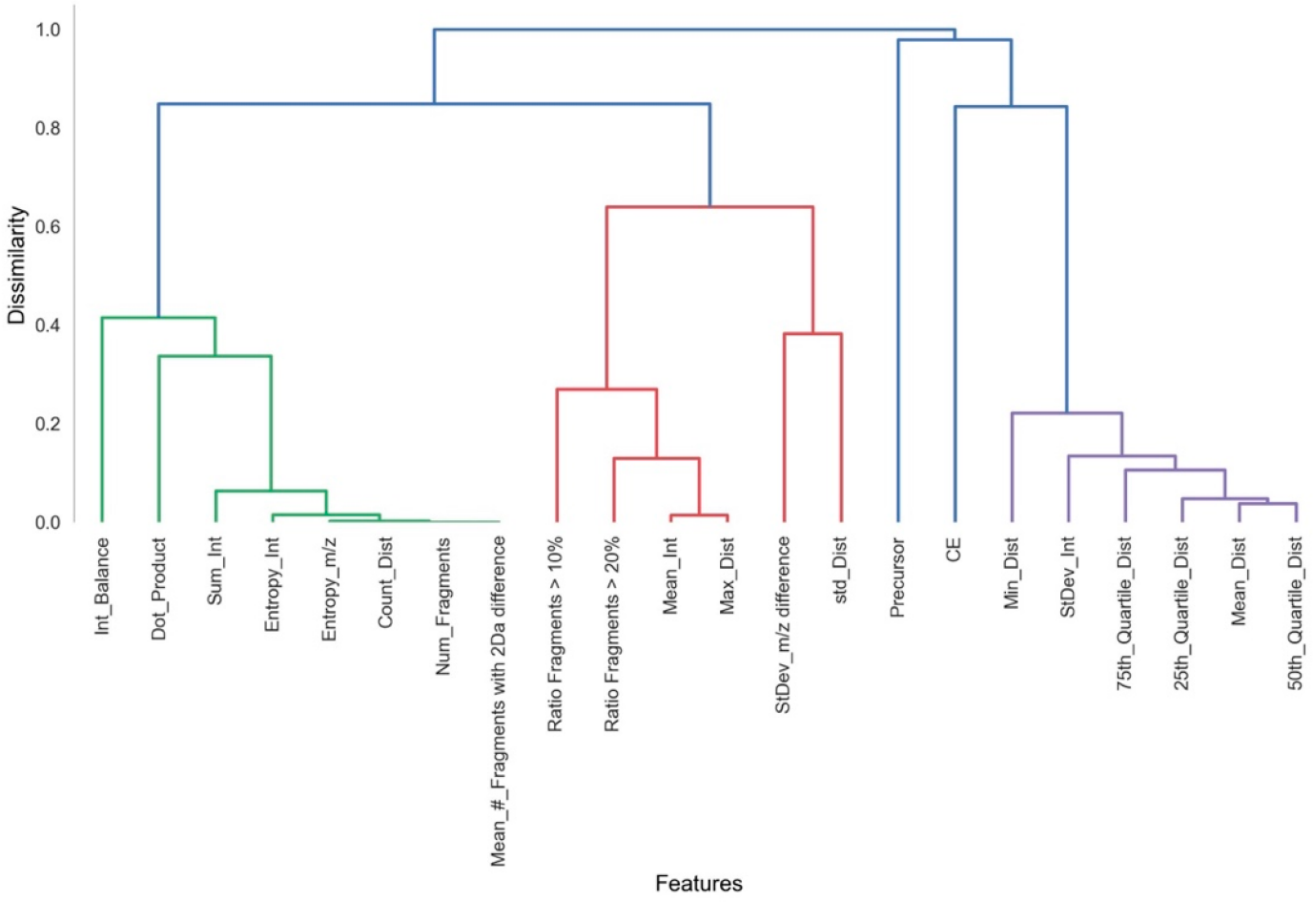
Hierarchical clustering dendrogram based on outcomes of the Spearman rank correlation test. The *y*-axis represents the degree of dissimilarity between the features, which is *D* = 1 – |ρ|, where ρ is the pairwise rank correlation coefficient. See Table S1 for all features.

A Spearman rank correlation test was applied to the combined *Handcrafted* and *Distance* features sets. As expected, the number of peaks and the count of distances are fully correlated (Figure 3). The two least correlated features were precursor *m*/*z* and CE Most distance features were all highly correlated and were clustered together. Features showing a dissimilarity score ≥0.3 were labeled as *uncorrelated* and were tested separately during the next step.

**Figure 4:**
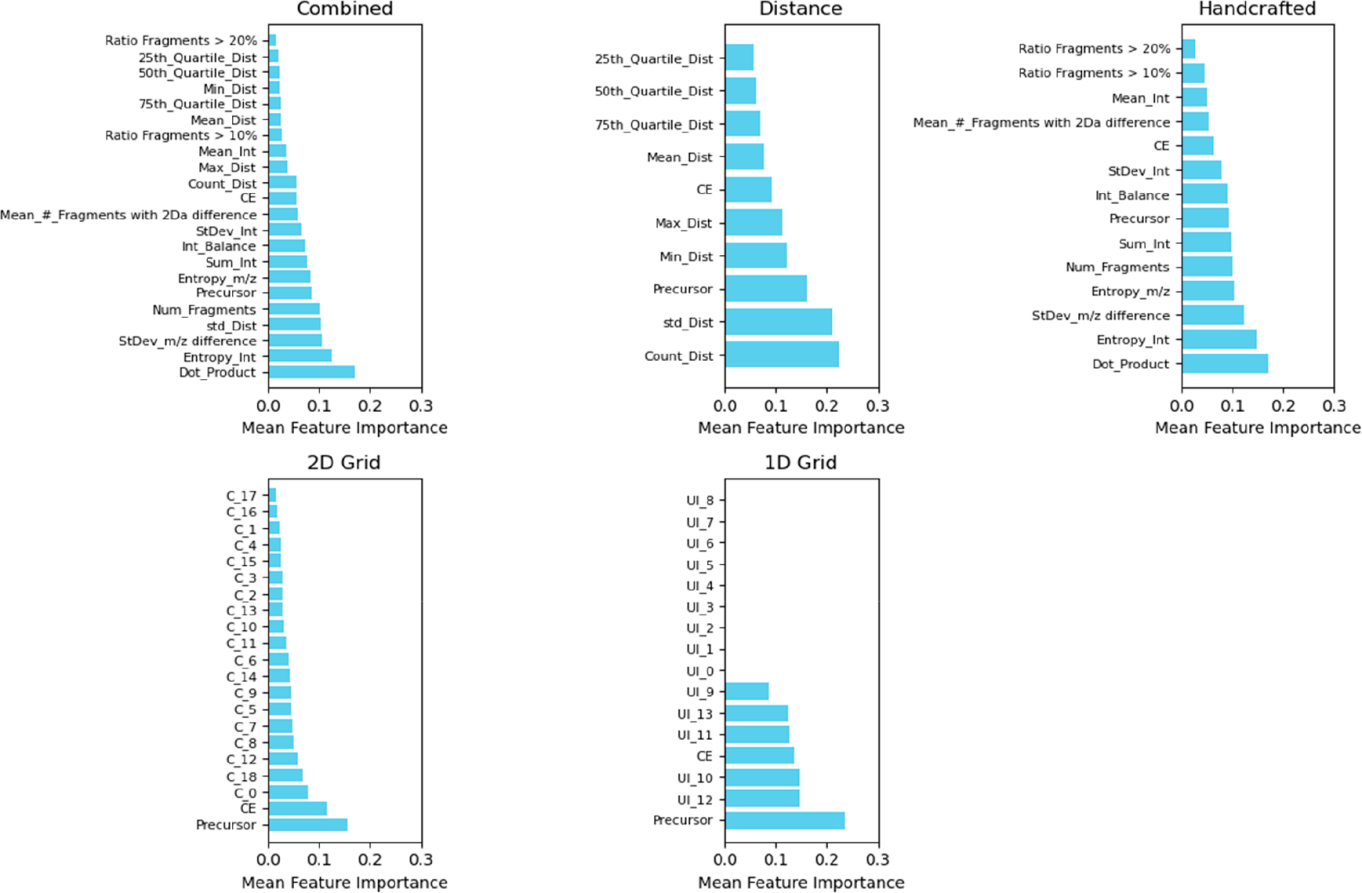
Mean feature importance for each set considered in the validation step. For 1D and 2D Grids, feature importance was calculated only for the optimum number of bins determined earlier (i.e., (14) and (19, 1), respectively). See Table S1 for all features.

After correlation testing, the feature importance was computed for each set (Figure 4). For *Distance* features, the most important were the count (i.e., number of fragments in the MS2), standard deviation of measured distances, and the precursor. For *Handcrafted* features, the most important were the dot product between *m*/*z* and intensities, the entropy of intensities, and the standard deviation of the differences between all *m*/*z* in the MS2. These features were also among the most important when considering the *Combined* set, although individually, *Distance* features seemed to have a higher importance compared to *Handcrafted* ones. Interestingly, the dot product was found to be the most important feature, suggesting that the alignment between *m*/*z* and the intensity is an important predictor of MS2 diagnostic information. Regarding *1D-* and *2D-Grids*, the precursor and CE were among the most important features, in particular for the *2D-Grid*. Interestingly, for the *2D-Grid*, features C0 and C18 were the most important features after the precursor and the CE, suggesting that the number of fragments at the extremities of the (normalized) MS2 is an important predictor of diagnostic information. For *1D-Grid*, only bins corresponding to higher intensities appeared to play a role in the classification, which is to be expected considering that the occurrence of intense (diagnostic) fragments was among the criteria used to discriminate between good and poor MS2 spectra.

**Figure 5:**
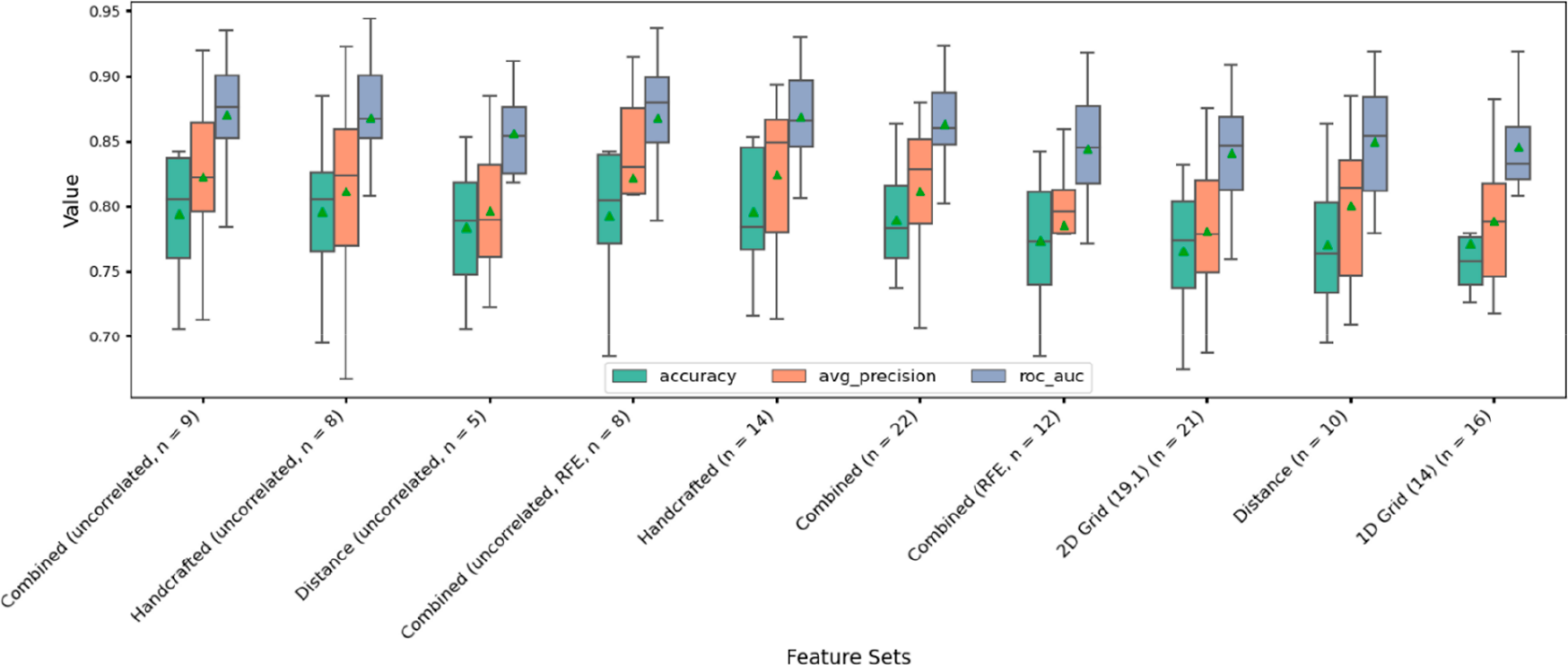
Validation performance results for combinations of feature sets. Either all features in the set were used or a subset thereof depending on the outcomes of the RFE and/or correlation analysis. *n* = number of features used. Combined = Handcrafted + Distance features combined. Uncorrelated = only features whose dissimilarity score was ≥0.3 were included. RFE = Feature set following recursive feature elimination (RFE). Feature sets were ordered from left to right by decreasing Log Loss value (not shown here, see Table S3).

Subsequently, RFECV was used for feature selection. While this method depends heavily on the model’s estimate of feature importance and is generally not safe as a feature selector alone, it is useful for creating new subsets of features that are evaluated in a separate procedure. In total, ten different feature groups were selected for evaluation (Figure 5), including the original feature sets, the *Combined* set, and the results of correlation testing and RFECV (applied together or separately). *2D-* and *1D-Grid* feature sets were computed using the number of bins giving the best performances (see Grid Feature Selection). *Uncorrelated* refers to feature subsets that passed through the correlation analysis (i.e., dissimilarity ≥ 0.3). Eventually, recursive feature elimination (RFE) was applied only to the *Combined* set given that none of the features were discarded for the other sets. As can be seen from Figure 5, the best performances were obtained for the *Combined* (i.e., *Handcrafted + Distance*) and *Handcrafted* feature sets, although overall performances were quite similar between all sets (detailed performances are reported in Table S3).

### 3.3 Testing Results

The most promising sets of features from each category (*Handcrafted, Distance* and their combination, *1D* and *2D-Grid*) based on validation results were compared using the holdout (test) set. For this purpose, all models were retrained using both training and validation sets. Results are reported in Table 1 and Figure S2. As can be seen, all feature sets perform reasonably well, achieving an average accuracy of about 83%, an average precision of 85%, and an ROC AUC of almost 91%. It is interesting to note that the baseline (i.e., number of peaks) had approximately 10% lower performances compared to the other feature sets. This might suggest that the number of peaks in MS2 spectra after normalization and noise removal is a rather good predictor of MS2 diagnostic information, yet the addition of other features substantially improves the classification. Regarding newly introduced *Distance* and *Grid* features, these showed results similar to the *Handcrafted* features derived from previous studies. These findings are also visible in Figure S2, which shows both the ROC and precision–recall curves. Unlike the baseline approach, the selected feature sets provided similar performances, especially with regard to the ROC curve. It is noteworthy that even though obtaining relevant features in the field of small molecules is more complex compared to proteomics, where one can rely on additional information/patterns due to the occurrence of repeating units (i.e., amino acids and peptides), results obtained here are consistent with performances reported in the literature. For instance, in the recent approach proposed by Gholamizoj and Ma,^9^ ROC AUC ranging from 68% to 89% were obtained for the classification of MS2 spectra of peptides.

**Table 1:** Performances (in %) of the Selected Models on the Test Set

Features	Accuracy	Average precision	ROC AUC	Number of Features
Handcrafted	84	87	92	14
Distance	83	87	91	10
Combined + RFE	83	84	90	8
1D Grid (14)	83	85	90	16
2D Grid (19, 1)	80	85	90	21
Baseline (no. peaks)	74	65	78	1

### 3.4 Optimized Model

Based on the outcomes of the testing step, the model with the best performance, namely, the one computed using the *Handcrafted* feature set, was further investigated and optimized. For this purpose, the confusion matrix of the RF classifier was directly examined instead of assessing the metrics derived from it. Using a standard threshold of 0.5, a precision of 77% was obtained. However, as discussed previously, it was decided to favor precision above the other performance parameters to minimize the chance of having a *poor* spectrum being mislabeled as *good*. For this purpose, the *f-beta* score^31^ was evaluated to find an optimal threshold for probability predictions. Using a *beta* parameter of 0.5, corresponding to a threshold of 0.666 and allowing a final accuracy of 84% to be obtained, a higher precision of 88% could be attained with a recall of 75%. Considering the purpose for which this model was developed, namely, to allow the selection of MS2-data providing diagnostic information for various applications, these results were in line with results from other quality prediction models developed in the field of proteomics.

## 4 Conclusion

Acquisition and processing of high-quality tandem mass spectra has clear advantages, both for identification and predictive modeling purposes. However, an automated approach to assess the diagnostic information on MS2 spectra in the field of (small) environmentally relevant molecules was still missing. In the context of this work, an RF classifier was trained to be capable of attaining comparable if not superior performances compared to approaches previously reported in the field of proteomics. The best performing model obtained in this work provided very similar results compared to the deep learning model recently developed by Gholamizoj and Ma^9^ (92% and 89% ROC AUC). Similar to the work done by Nesvizhskii et al.,^11^ the classifier was not affected by the presence of potentially correlated features. With respect to results obtained using the *Grid* features-based model, the RF classifier obtained here using a 1D grid outperformed the Gaussian Mixed model developed by Logan et al.^25^ (89% versus 76% ROC AUC, respectively). Similarly, the model developed here also performed slightly better compared to the one obtained through boosting when using a 2D grid (87% versus 85% ROC AUC, respectively). Despite being developed on a rather small data set, results suggest that the tested features and the optimized classifier could be a very useful tool to automatically classify MS2 spectra of environmentally relevant compounds based on the quality of their diagnostic information. Applications could range from improving and automating spectral library curation and identification, prioritization of features for further identification in NTS applications, improve performances of MS2-based computational methods, and even acquisition, should these approaches become part of acquisition parameters in DDA methods for instance. In the future, the model’s performances should be evaluated on a larger data set and/or develop more advanced models (e.g., deep learning). For instance, convolutional neural network could be trained using images of MS2 spectra obtained using the proposed 1D- or 2D-Grids. Moreover, the proposed model should be evaluated to determine whether it allows increasing identification/discovery rates in non-target screening applications for environmental analysis. Finally, moving away from a binary approach by introducing multiple classes or developing a continuous score based on the model’s outputs could be promising directions to further advance this approach.

## Supporting Information Available

Accuracy, precision, and ROC AUC obtained using a 1D unevenly distributed grid (Figure S1) and ROC curves and precision–recall curves obtained for the models trained using the selected feature sets (Figure S2); summary of all features and sets used to train the RF classifier (Table S1), bin number combinations providing the best metrics for 2D grids (Table S2), and details of validation performances of the computed feature sets (Table S3) (PDF)
