# Supplementary material for: Predicting the Diagnostic Information of Tandem Mass Spectra of Environmentally Relevant Compounds Using Machine Learning
Source: Anal Chem. 2023 Oct 9;95(42):15810–7. doi: 10.1021/acs.analchem.3c03470 (PMC10603772; doi:10.1021/acs.analchem.3c03470)
Supplement: Supplementary file 1 — ac3c03470_si_001.pdf [file ac3c03470_si_001.pdf]

SUPPORTING INFORMATION

Predicting the diagnostic information of tandem mass spectra of environmentally relevant compounds using machine learning

[1]Codrean S., [1]Kruit B., [2]Meekel N., [2]Vughs D., [3,2]Béen F.*

1) Faculty of Science, Artificial Intelligence, Vrije Universiteit Amsterdam, Amsterdam, The Netherlands
2) KWR Water Research Institute, Nieuwegein, the Netherlands
3) Chemistry for Environment and Health, Amsterdam Institute for Life and Environment (A-LIFE), Vrije Universiteit Amsterdam, Amsterdam, The Netherlands

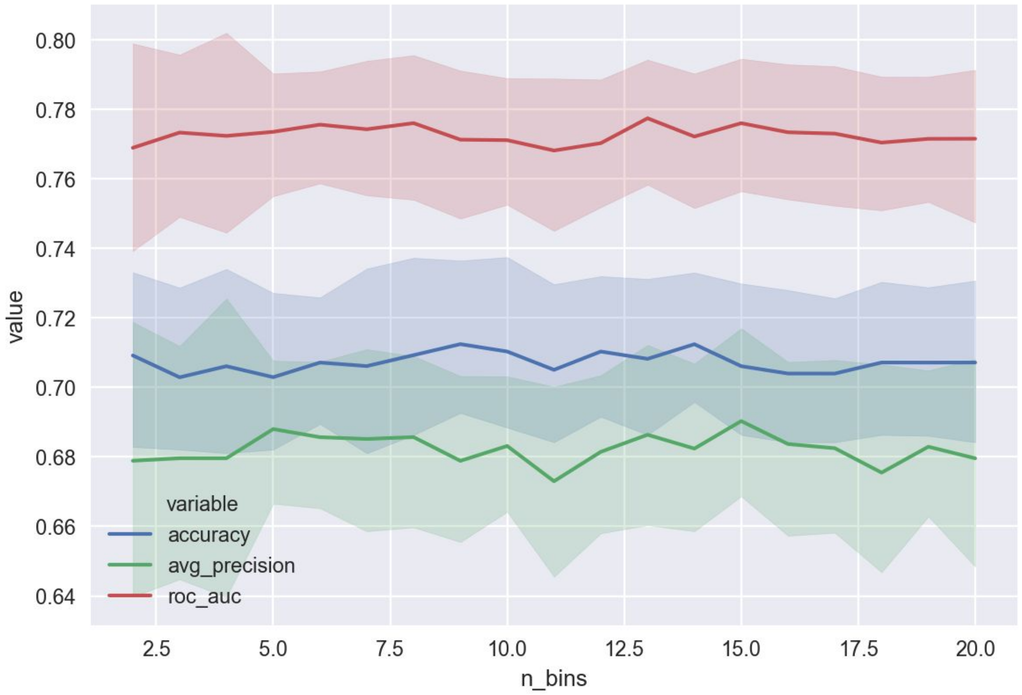

Figure S1: Accuracy, precision and ROC AUC obtained using a 1D unevenly distributed grid.

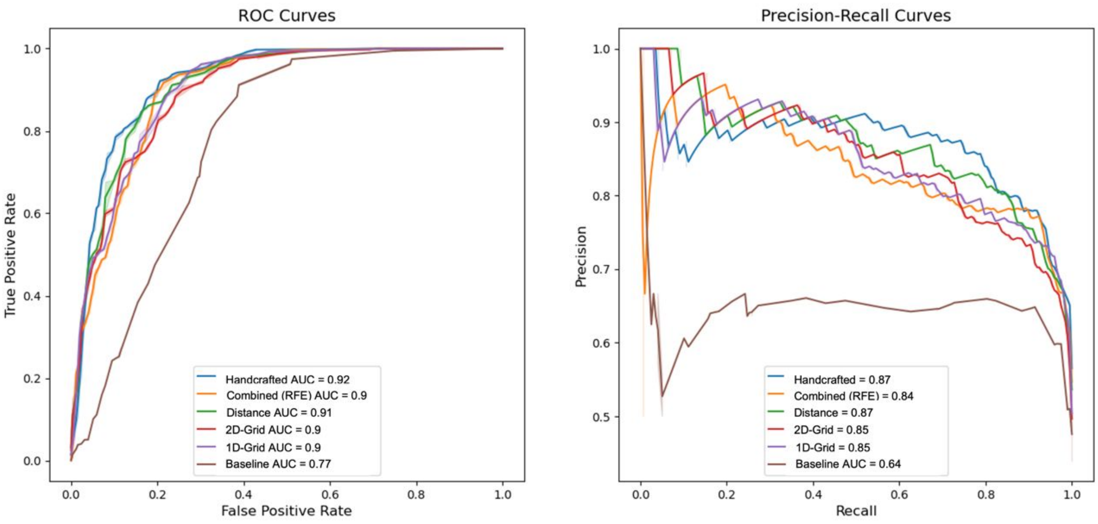

Figure S2: ROC curves (left) and Precision-Recall curves (right) obtained for the models trained using the selected feature sets.

Table S1: Summary of all features and sets used to train the RF classifier.

Feature set	Feature name	Description
Precursor	Precursor	m/z of the precursor ion.
Collision energy	CE	Collision energy used to fragment the precursor ion.
Distance	Count_Dist	Number of elements in the distance vector, equivalent to the number of fragments in the MS2. The distance vector was computed as the Euclidean distance between the centroid and each fragment in the spectrum.
	Mean_Dist	Mean of the distance vector.
	Stdev_Dist	Standard deviation of the distance vector.
	Min_Dist	Minimum of the distance vector.
	Max_Dist	Maximum of the distance vector.
	25^{th}_Quartile_Dist	First quartile of the distance vector.
	50^{th}_Quartile_Dist	Second quartile of the distance vector.
	75^{th}_Quartile_Dist	Third quartile of the distance vector.
Handcrafted	Num_Fragments	Number of fragments in the MS2, equivalent to Count_Dist
	Stdev_Int	Standard deviation of intensities.
	Dot_Product	Dot product between the m/z and intensity vectors.
	Ratio Fragments > 10%	Ratio of fragments with relative intensity above 10%
	Ratio Fragments > 20%	Ratio of fragments with relative intensity above 20%
	Stdev_m/z difference	Standard deviation of the difference between consecutive m/z.
	Mean_#_Fragments with 2 Da difference	Mean number of fragments having a difference of 2 Daltons.

	Int_Balance	Intesity balance calculated by dividing the *m/z* axis into a number of bins of equal width and subtracting the total intensity of the first bin from the sum of the intensities of the remaining bins.
	Entropy_m/z	Shannon entropy of the m/z vector calculated using the *entropy* function of *scipy.stats* module of Python.
	Entropy_Int	Shannon entropy of the intensity vector calculated using the *entropy* function of *scipy.stats* module of Python.
1D-Grid	Bins (n)	Number of unevenly distributed bins used to separate the intensity axis.
2D-Grid	Bins (n, m)	Number of bins used to separate the m/z and intensity axes.

Table S2: Bin number combinations providing the best metrics for 2D Grids. Results are sorted by log loss and decreasingly by average precision, ROC AUC and accuracy. Standard deviations are shown between brackets.

(# m/z bins, # intensity bins)	Accuracy		Average Precision		ROC AUC		Log Loss	
(19, 1)	75	(5)	73	(6)	81	(4)	8.77	(1.56)
(10, 1)	74	(3)	69	(6)	79	(4)	9.06	(1.03)
(12, 1)	73	(5)	74	(6)	80	(5)	9.28	(1.65)
(2, 11)	73	(4)	70	(9)	79	(6)	9.28	(1.49)

Table S3: Details of validation performances of the computed feature sets. Standard deviations are shown between brackets.

**Features**	**Accuracy**	**Average Precision**	**ROC AUC**	**Log Loss**	**Number of Features**
Combined (uncorrelated)	80 (5.0)	82 (7.4)	87 (4.4)	7.2 (1.8)	9
Handcrafted (uncorrelated)	80 (4.8)	83 (5.7)	87 (4.1)	7.2 (1.73)	8
Distance (uncorrelated)	79 (4.1)	80 (5.0)	86 (3.6)	7.4 (1.49)	5
Combined (uncorrelated, RFE)	79 (4.9)	83 (6.7)	87 (4.4)	7.5 (1.76)	8
Handcrafted	79 (3.7)	82 (6.3)	86 (4.2)	7.4 (1.36)	14
Combined	79 (4.3)	81 (6.5)	86 (4.8)	7.5 (1.57)	22
Combined (RFE)	78 (5.8)	79 (5.7)	85 (4.1)	7.7 (2.11)	12
2D-Grid (19,1)	77 (3.0)	79 (5.6)	86 (3.5)	7.9 (1.1)	21
Distance	77 (5.1)	80 (6.3)	85 (4.4)	8.0 (1.86)	10
1D-Grid (14)	77 (4.8)	79 (4.5)	85 (3.2)	8.1 (1.73)	16
